# The Availability of a Functional Tumor Targeting T-Cell Repertoire Determines the Anti-Tumor Efficiency of Combination Therapy with Anti-CTLA-4 and Anti-4-1BB Antibodies

**DOI:** 10.1371/journal.pone.0066081

**Published:** 2013-06-13

**Authors:** Benjamin A. H. Jensen, Sara R. Pedersen, Jan P. Christensen, Allan R. Thomsen

**Affiliations:** University of Copenhagen, Faculty of Health and Medical Sciences, Department of International Health, Immunology and Microbiology, Blegdamsvej 3C, Copenhagen, Denmark; Carl-Gustav Carus Technical University-Dresden, Germany

## Abstract

It has previously been found that combination therapy with anti-CTLA-4 and anti-4-1BB antibodies may enhance tumor immunity. However, this treatment is not efficient against all tumors, and it has been suggested that variations in tumor control may reflect differences in the immunogenicity of different tumors. In the present report, we have formally tested this hypothesis. Comparing the efficiency of combination antibody therapy against two antigenically distinct variants of the B16.F10 melanoma cell line, we observed that antibody therapy delayed the growth of a variant expressing an exogenous antigen (*P*<0.0001), while this treatment failed to protect against the non-transfected parental line (*P* = 0.1850) consistent with published observations. As both cell lines are poorly immunogenic in wild type mice, these observations suggested that the magnitude of the tumor targeting T-cell repertoire plays a major role in deciding the efficiency of this antibody treatment. To directly test this assumption, we made use of mice expressing the exogenous antigen as a self-antigen and therefore carrying a severely purged T-cell repertoire directed against the major tumor antigen. Notably, combination therapy completely failed to inhibit tumor growth in the latter mice (*P* = 0.8584). These results underscore the importance of a functionally intact T-cell population as a precondition for the efficiency of treatment with immunomodulatory antibodies. Clinically, the implication is that this type of antibody therapy should be attempted as an early form of tumor-specific immunotherapy before extensive exhaustion of the tumor-specific T-cell repertoire has occurred.

## Introduction

Following the overwhelming success of immunomodulatory antibodies in the treatment of autoimmune diseases, it is now time to fully exploit the potential of this class of potent drugs in the treatment of cancer. Several antibodies are already in clinical use, while others are under investigation in pre-clinical studies [1]–[3]. In this regard, antibodies against co-stimulatory molecules, such as cytotoxic T lymphocyte antigen 4 (CTLA-4) and 4-1BB, have emerged as potentially important therapeutics against various tumors [4], [5].

CTLA-4 is a co-inhibitory receptor expressed on T-cells shortly after their activation [6], and it has been found to play an important role in the modulation of antigen-specific immune responses. In addition, expression of CTLA-4 is critical to the functionality of regulatory T-cells (Tregs) *in vivo* [7]. Collectively, blocking of this molecule allows for efficient stimulation of immune responses towards weak antigens, such as tumor antigens; however, it also increases the risk of self-reactivity, and studies in murine models have underscored this risk. Perhaps more importantly, autoimmune manifestations have also been observed in human patients [8], and careful clinical management is essential, if immune-related toxicities are to be kept acceptable [9].

4-1BB is a molecule belonging to the tumor necrosis superfamily. It is transiently up-regulated on T-cells subsequent to activation, and ligand binding is known to augment CD8 T-cell activity [10], [11]. In various tumor models, agonistic anti-4-1BB antibodies have been found to improve tumor control [10]. Interestingly, even though 4-1BB signaling may render effector T-cells resistant to the inhibitory effect of Tregs [12], treatment with anti-4-1BB antibodies has also been found to reduce autoimmunity in lupus-prone mice [13].

Since agonistic anti-4-1BB antibodies appear to both improve anti-tumor responses and, in some cases, reduce autoimmunity, it has been suggested to combine this treatment with antibodies blocking CTLA-4 [4], [5]. In fact, a study published by Kocak et al. seems to provide proof-of-concept in this respect [5]. Thus, these authors examined the efficacy of this combinatorial regimen in two distinct tumor models; MC38 colon carcinoma cells and B16 melanomas. Interestingly, they found that only MC38 challenged mice were significantly protected. As a plausible explanation for this, it was suggested that the difference in clinical effect might result from differences in the intrinsic immunogenicity of the tested tumor cell lines. Yet, as only two very different tumor cell lines were studied, this explanation together with its implications could not be scientifically verified. Considering the clinical importance of developing new combinational treatments of human cancers, we decided to revisit the above subject and formally test whether the anti-tumor potential of combining these antibodies is in fact limited by the intrinsic immunogenicity of the involved tumor cells or whether it is more the availability of a functionally intact, tumor-specific T-cell repertoire, which is critical.

Accordingly, we made use of two closely related cell lines: wild type (WT) B16.F10 cells and a gene modified variant, B16.F10-GP, expressing the immunodominant epitope of the glycoprotein (GP) of lymphocytic choriomeningitis virus (LCMV) [14], [15]. WT B16.F10 cells are poorly immunogenic, in part, due to weak MHC class I expression and a nonexistent MHC class II expression [16]. In contrast, B16 variants expressing exogenous transgenes are quite antigenic despite poor intrinsic immunogenicity, and for this reason they are commonly used to monitor the efficiency of otherwise, e.g. vaccine, induced anti-tumor immune responses [4], [14], [17].

As an additional tool, we employed a mouse strain (Alb-1) expressing LCMV-GP as a self-antigen under the albumin promoter [18]. In these mice, the GP-specific CD8 T-cell repertoire is severely depleted, and, as a consequence, GP-specific responses in Alb-1 mice are greatly reduced compared to those induced in their WT counterparts [18].

Using the described experimental approach, we find that differences in the protective capacity of combinatorial therapy with antagonistic anti-CTLA-4 and agonistic anti-4-1BB antibodies do not so much reflect differences in the intrinsic immunogenicity of the tumor cells as the availability of a functionally intact T-cell repertoire targeting antigens expressed by the tumor cells.

## Materials and Methods

### Ethics Statement

Experiments were conducted in accordance with national Danish guidelines (Amendment #1306 of November 23, 2007) regarding animal experiments as approved by the Danish Animal Inspectorate, Ministry of Justice, permission #2011/561–87.

### Mice

Alb-1-GP transgenic C57BL/6 mice were the progeny of breeding pairs originating from the animal facility of Spital, Zürich [18], and kindly provided by Daniel Pinschewer. WT C57BL/6 mice were purchased from Taconic M&B (Ry, Denmark).

### Tumor Cell Lines

B16.F10 and B16.F10-GP (expressing the minimal epitope of the LCMV glycoprotein, GP33-41) melanoma cells were cultured in DMEM 1965 supplemented with 10% FCS, glutamine, streptomycin, and penicillin. Additionally, B16.F10-GP cells were grown in the presence of G418 (0.8 mg/ml). Both cell lines were kind gifts from Hanspeter Pircher, Germany [14].

### Antibodies

Agonistic anti-4-1BB stimulating monoclonal antibody (mAb) from 3H3 hybridomas [11] and anti-CTLA-4 mAb from 9H10 hybridomas [19] were purified from cell culture supernatant using a protein G column.

### Adenoviral Vector

Replication deficient E1-deleted Ad5 vector with a non-functional E3 gene expressing GP of LCMV linked to the murine invariant chain (Ii), designated Ad5-IiGP, was produced as described previously [20].

### Injections and Tumor Measurements

All mice were subcutaneously injected with 10^6^ melanoma cells in the right flank at day 0. The mice were shaved at the injection site prior to inoculation. When the tumors reached the size of ≥12 mm, the mice were euthanized for ethical reasons. The tumor volumes were calculated as length×width^2^×0.5236. When relevant, vaccinations with human Ad 5-based vectors were administered in the right hind footpad 5 days post tumor inoculation.

### Statistical Analysis

Comparison among groups in the survival experiments was analyzed by the log-rank test (Mantel-Cox). Tumor volume are presented as mean ± s.d. and analyzed by 2way ANOVA. Prism 6, GraphPad software (GraphPad Software Inc.) was used for all statistical analysis. *P*<0.05 was considered statistically significant.

## Results and Discussion

### Treatment with Agonistic Anti-4-1BB and Blocking Anti-CTLA-4 Antibodies Augments Tumor Control

We have previously shown that the growth of B16.F10-GP tumors can be partially controlled by therapeutic vaccination with Ad5 vectors expressing GP [17]. We could also demonstrate that tumor control was markedly improved if GP was tethered to the MHC class II associated invariant chain (Ii). However, under the conditions we normally used, we very rarely observed any long-term survivors, and for this reason we have been searching for treatment modalities, which in combination with our vaccine would result in improved long-term tumor control. Different immunomodulatory antibodies have been tested with varying success [21]. In the context of these studies, we decided to test a combination of agonistic anti-4-1BB with blocking anti-CTLA-4. This regimen has been described by Kocak et al. to both enhance cancer therapy and reduce autoimmunity in a murine carcinoma model [5]. Interestingly, the antibody treatment they used failed to protect B16.F10 challenged mice. Nevertheless, we hypothesized that by also targeting B16.F10 melanomas through antigen-specific vaccination, a clinically relevant improvement would be observed.

**Figure 1:**
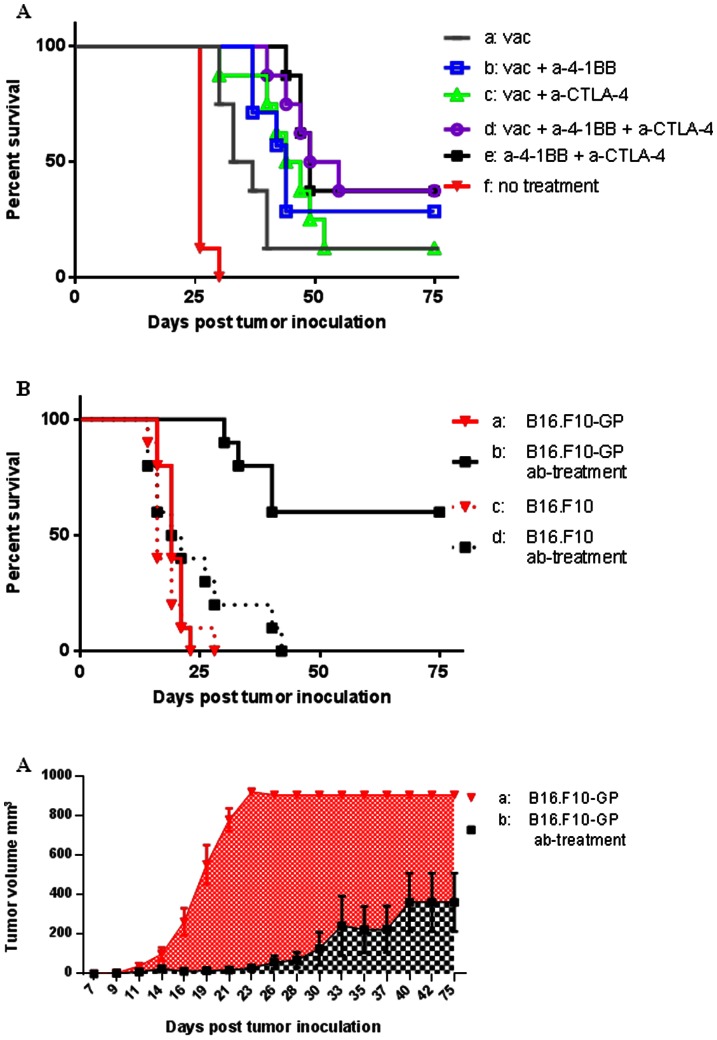
Treatment with agonistic anti-4-1BB and blocking anti-CTLA-4 delays tumor growth only in mice challenged with tumor cells expressing a strong foreign antigen. Mice (n = 8–10 mice/group) were inoculated s.c. in the right flank with 10^6^ melanoma cells at day 0 and antibodies were administered i.p. as described. A: a) Mice vaccinated with 2×10^7^ IFU Ad-IiGP in the right hind footpad 5 days post B16.F10 inoculation (pt). b) As in a, plus treatment with 200 µg anti-4-1BB at days 9 and 12 pt. c) As in a, plus treatment with 100 µg anti-CTLA-4 at day 5 and 50 µg anti-CTLA-4 at days 7 and 9 pt. d) As in a, plus treatment with anti-4-1BB and anti-CTLA-4 as in b and c, respectively. e) B16.F10 tumor cells, no vaccination, antibody treatment as in d. f) B16.F10 tumor cells, no treatment. P-values: e vs. f <0.0001; a vs. d = 0.0220; d vs. e = 0.9400. B: Solid lines indicate mice challenged with B16.F10-GP cells; dashed lines indicate mice challenged with B16.F10 cells. Grey lines indicate mice that did not receive any treatment. Black lines indicate mice treated i.p. with 200 µg anti-4-1BB at days 9 and 12 plus 100 µg anti-CTLA-4 at days 5 and 50 µg anti-CTLA-4 at days 7 and 9 pt. Mortality of tumor bearing mice as a function of time. P-values: a vs. b <0.0001; a vs. d = 1.0000; b vs. d = 0.0003; c vs. d = 0.1850 C: Tumor volumes as a function of time; data are presented as mean ± SEM. P-values: a vs. b <0.0001.

When mice were challenged with B16.F10-GP, followed by antigen-specific vaccination and immune modulatory antibody treatment, we observed significantly improved tumor control compared to mice given no antibody treatment (*P* = 0.0220– Figure 1A). Hence, at first sight, the antibody treatment seemed to significantly increase the protective potential associated with antigen-specific vaccination. However, to our surprise unvaccinated mice, which only received the antibody treatment were equally well protected (*P* = 0.9400), indicating that the antibody treatment sufficed for a marked clinical effect.

### Gene Modified B16.F10 Cells are much more Susceptible to Antibody-induced Tumor Control than their WT Counterparts

As our results seemingly conflicted with those of Kocak et al. [5], describing that this antibody combination was insufficient for treatment B16 tumors, we hypothesized that the success of this antibody treatment relates to the presence of a “strong” foreign antigen on the tumor cells combined with a substantial population of matching T-cells. This would not be the case for WT tumor cells, which would not be expected to express any strong antigens.

To test this prediction, we injected WT mice with 10^6^ B16.F10 cells, with or without expression of exogenous antigen (GP), followed by antibody treatment. The results presented in Figure 1B–C clearly support our hypothesis: antibody treatment did not significantly impact tumor growth in B16.F10 challenged mice (*P* = 0.1850); whereas the effect on B16.F10-GP challenged mice were highly significant (*P*<0.0001). Untreated mice were equally susceptible to both cell lines (*P* = 1.000).

### The Antibody-induced Control of B16.F10-GP Cells is Abolished in Mice Carrying GP as a Self-antigen

The observed difference in the efficiency of antibody therapy against tumor cells bearing exogenous antigen versus no antigen underscored a role for functionally intact, tumor-specific T-cells in the antibody induced tumor control. Although overall T-cell depletion of course would validate a role of T-cells, this treatment would not provide any information regarding the fine specificity requirements of the involved cells. For this reason, we decided to use a subtler approach to test whether successful antibody induced tumor control requires a functionally intact population of tumor-specific CD8 T-cells. Thus, we used Alb-1 mice, which express GP as a self-antigen and for that reason have a severely depleted T-cell repertoire for this antigen compared to WT mice [18].

**Figure 2:**
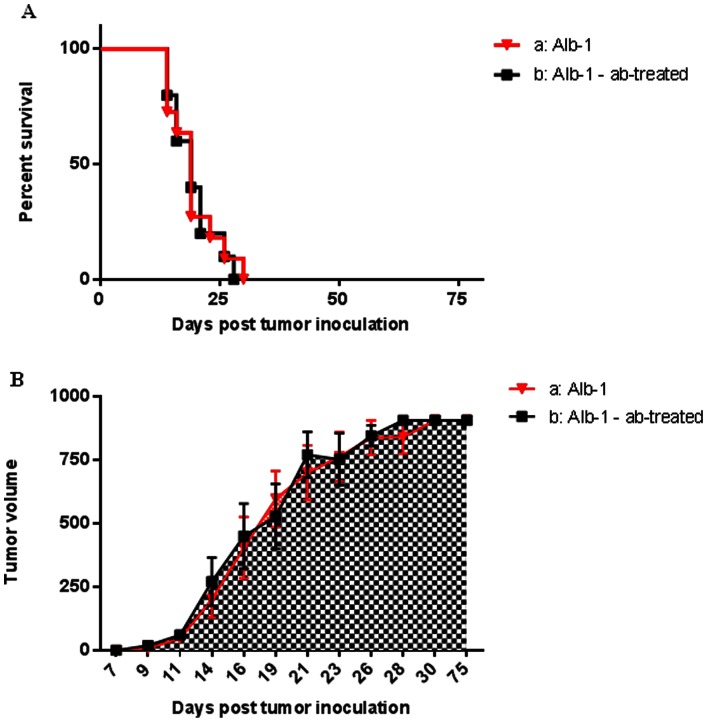
Combined antibody therapy with agonistic anti-4-1BB and anti-CTLA-4 antibodies does not delay growth of B16.F10-GP tumors in GP tolerant Alb-1 mice. Mice (n = 10-11 mice/group) were inoculated s.c. in the right flank with 10^6^ B16.F10-GP melanoma cells at day 0. Grey lines indicate mice that did not receive any treatment. Black lines indicate mice treated i.p. with 200 µg anti-4-1BB at day 9 and 12 plus 100 µg anti-CTLA-4 at day 5 and 50 µg anti-CTLA-4 at day 7 and 9 pt. A) Mortality of tumor bearing mice as a function of time. P-values: a vs. b = 0.8740 B) Tumor volumes as a function of time; data are presented as mean ± SEM. P-values: a vs. b = 0.9992.

As predicted, if an intact tumor targeting T-cell population play a decisive role when it comes to the efficiency of this antibody combination, Alb-1 mice, unlike WT mice, were equally susceptible to tumor challenge with B16.F10-GP cells whether they received antibody therapy or not (*P* = 0,8584 – Figure 2).

### Concluding Remarks

The results of the present study confirm that this antibody combination is inefficient against the WT B16 cell line. In contrast, the antibodies could efficiently delay the growth of a variant of this tumor cell line expressing a foreign antigen. Since the two cell lines are identical except for the presence of the transgene, and there is clear evidence in the literature that neither of these cell lines are very immunogenic when inoculated into normal WT mice [15], our results strongly indicate, that availability of tumor-targeting T-cell repertoire represents a key factor in deciding the clinical efficiency of combination therapy with anti-4-1BB and anti-CTLA-4. Furthermore, the efficiency of antibody treatment clearly relates to the size of the T-cell repertoire targeting antigens expressed by the tumor cells. Thus, in mice (Alb-1) with a T-cell repertoire purged of most T-cells specific for the major tumor antigen relevant under the current test conditions (GP) [18], combined antibody treatment did not significantly delay the growth of GP-expressing tumor cells. Some might argue that it is self evident that the presence of tumor-targeting T-cells represents a precondition for immunmodulatory antibodies like anti-CTLA-4 and anti-4-1-BB to have an effect on tumor growth. However, this insight is often ignored or forgotten in actual clinical practice. Thus, since cancer immunoediting is assumed to rapidly remove the most immunogenic cancer cells [22], the present findings serve to underscore that this antibody combination therapy is likely to be efficient only in cases where the remaining low-immunogenic tumor cells express tumor antigens for which there is little or no purging of the naïve T-cell repertoire, i.e. neoantigens representing mutated self or viral antigen. Furthermore, as prolonged cancer growth is believed to be associated with functional impairment of relevant T-cells [23], [24], results like ours tend to imply that immunomodulatory antibody treatment should be tested as an early treatment modality – before T–cell exhaustion is severely progressed - and not as a last resort.
